# Professional Fees

**Published:** 1900-12

**Authors:** 


A Monthly Review of Medicine and Surgery.

EDITOR:
WILLIAM WARREN POTTER, M. D.

All communications, whether of a literary or business nature, books for review and exchanges should be addressed to the editor: 284 FRANKLIN STREET, BUFFALO, N. Y.

VOL. XL.—LVI. DECEMBER, 1900. No. 5.

### PROFESSIONAL FEES.

DURING the last month a case has been on trial in Buffalo which required several local physicians to give their opinions as to the value of medical services. Their testimony was commented upon rather sharply by some of the local papers and, afterward, "a physician" expressed his views in a letter in the open column. If the reports of the testimony were correct, as given by the doctors, it is evident that this very practical subject will bear a little further consideration along the same lines even in a medical journal.

One point which was made rather nebulous to the jury was the question why one amount should be charged to the poor and another to the rich for the same operation. It is probably true that the circumstances of the patients sometimes are factors in determining the fee.

The fact is, the doctor has to give a large part of his time and energy for nothing, in hospitals, or study, before the rich ask for his services at all, and what they really pay for, is not alone the service rendered just then, but for their proportional part of his time which was spent in gaining adequate knowledge. It is perfectly true also that the rich make greater demands upon our time and attention, and some of them look upon the physician whom they employ as rather akin to a well-educated servant.

When such patients enter an office, they expect to be seen promptly, to be listened to patiently, no matter how long or how irrevelant may be their story, and to have special care bestowed upon them. Or, when visited at their homes, ills that are trifles to others are of importance to them, because unaccustomed to annoyances and the physician is often obliged to dance attendance and cater to peculiarities as he would not, unless paid for it.

Most physicians will probably agree as to the justice of thus varying their charge according to the circumstances of the patient. But, on the other hand, they differ not a little when it comes to another aspect of this question—namely, the different fees charged by different doctors for the same service to the same individual. Thus, nearly every county has its so-called "fee bill" which is usually approved with some ceremony at a meeting of the local association, and then disregarded except as a general plan of guidance. A few may cry out against lack of uniformity, but the real fact is, that other things being equal in a reputable medical practice, as in every other branch of business, the amount realised depends upon the amount of time and capital invested.

If one has had no early training and has barely succeeded in passing his examination after the usual three or four years' course, has purchased a dozen or twenty books and read but few of them, the professional investment of such a man is exceedingly small as compared with that of another, who after taking his academic course has graduated with honor, served one or two years in a hospital, spent several thousand dollars in books and instruments, and worked for years, often without pay, that his experience might be larger and his judgment more mature. It seems only natural that this much larger investment of time and capital should give a proportionately larger return to the second than to the first. The fresh graduate who is paid $25.00 for his first case of appendicitis, receives more in proportion than does the careful and experienced surgeon at ten times that fee.

The general impression of doctor's fees, is often suggestive of the reply given by a farmer who asked for his bill at a fashionable hotel. The smart clerk promptly told him the amount without looking at the books, whereupon the old man straightened up and said, "guess again, young feller, I've got more money in my pocket'n that." It is worth while to remember that doctors do not guess at such fees as they can hope to get, but they earn what they receive, often with no small expenditure of labor and care and anxiety for their patients.

